# Multiple Small Coronary Artery Fistulas Emptying into the Left Ventricle: A Rare but Challenging Problem

**DOI:** 10.1155/2016/2406250

**Published:** 2016-07-25

**Authors:** Omar Kahaly, Konstantinos Dean Boudoulas

**Affiliations:** Department of Medicine, Division of Cardiovascular Medicine, The Ohio State University Wexner Medical Center, Columbus, OH 43210, USA

## Abstract

A coronary artery fistula (CAF) is an abnormal communication between a coronary artery and a cardiac chamber or a great vessel. CAFs are rare based on coronary arteriography and when found they most often empty into the right ventricle and atrium and less often into the high pressure, low compliance left ventricle (LV). A patient who presented with atypical chest pain and was found to have multiple small CAFs originating from the ramus intermedius coronary artery and emptying into the LV is presented. This case highlights the challenges in providing an appropriate therapy for multiple small CAFs emptying into the LV.

## 1. Introduction

Coronary artery fistula (CAF) is defined as an abnormal communication between one or more coronary arteries and a cardiac chamber or a great vessel. Abbott more than a century ago has described CAFs emptying into a cardiac chamber on autopsy studies in 1908 [1]. The pathogenesis of a CAF has been speculated to be secondary to a partial persistence of sinusoidal connections between the lumens of the primitive tubular heart that supply myocardial blood flow in the early embryologic period. Normally, the intramyocardial sinusoids regress and persist only as Thebesian vessels in adults; however, if this regression fails, abnormally prominent fistulous communication persists between the coronary arteries and a cardiac chamber [2]. Based on coronary arteriography, the incidence of a CAF is 0.1% [3]. The majority of CAFs are congenital in origin, but they can also be acquired after trauma or a cardiac procedure (e.g., endomyocardial biopsy, pacemaker or defibrillator placement, percutaneous coronary intervention, and cardiac surgery). In addition, CAFs have been thought to occur in severe coronary atherosclerosis due to aberrant neovascularization in which collateral vessels inadvertently terminate into a cardiac chamber [4].

Most often CAFs empty into the right ventricle, followed by the right atrium, the pulmonary artery, and then coronary sinus (low pressure, high compliance chambers), and less often empty into the high pressure, low compliance left ventricle (LV) [2, 3, 5]. The major determinants of blood flow through the CAF are related to hemodynamic factors (i.e., coronary artery pressure; compliance and pressure of the receiving chamber) and the size of the CAF. A patient with multiple small CAFs originating from the ramus intermedius coronary artery and emptying into the LV is presented.

## 2. Case Report

**Figure 1:**
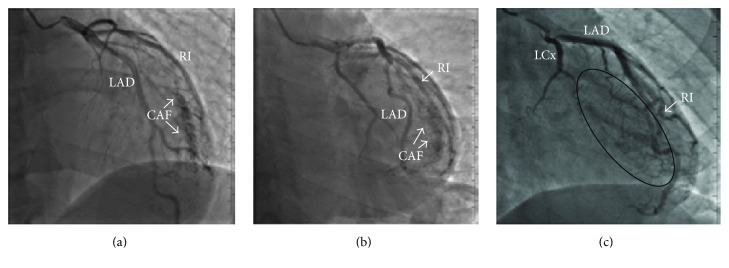
(a, b) Coronary arteriography demonstrating coronary artery fistulas (CAFs) arising from the ramus intermedius (RI) coronary artery and emptying into the left ventricle (LV). (c) The black circle shows filling of the LV chamber from the CAFs that arise from the RI seen during coronary arteriography. Left anterior descending (LAD) artery; left circumflex (LCx) artery.

A 47-year-old male with no significant past medical history or family medical history was admitted to the hospital with an ongoing complaint of fatigue and chest pain. The patient described the chest pain as dull and sharp in quality that was constant and located over the left side of his chest radiating to his left shoulder. The patient denied a history of syncope or presyncope. On physical examination the heat rate was 40 beats per min and blood pressure was 115/56 mmHg. The heart sounds were normal; there were no gallops, murmurs, or clicks. Admission electrocardiogram demonstrated sinus bradycardia with 40 beats per minute and no evidence of ischemia, myocardial injury, prior myocardial infarction, or other abnormalities. Exercise echocardiogram did not reveal evidence of ischemia; however, target heart rate was not achieved. For further evaluation of the chest pain, coronary arteriography was performed showing no evidence of coronary atherosclerosis, but multiple small CAFs arising from the ramus intermedius coronary artery and emptying into the LV were seen (Figure 1). To define a possible cause of sinus bradycardia, an electrophysiology study was performed; bradycardia was attributed to a high vagal tone that appropriately responded to isoproterenol infusion. Cardiac magnetic resonance imaging was within normal limits and there was no evidence of myocardial edema, inflammation, scarring, or infiltration. Thyroid function tests were also normal. Since it was thought that the patient's symptoms were not related to myocardial ischemia, he was discharged without therapy for the CAFs and at 6-month follow-up he was doing well without the recurrence of chest pain.

## 3. Discussion

Often patients with CAFs are asymptomatic; however, common symptoms that may develop include chest pain, dyspnea, and palpitations. Depending on the size and blood flow through the CAF, a murmur may be heard on auscultation; a continuous murmur is reported more often than a systolic or diastolic murmur [6]. When the amount of blood through the CAF is small, a cardiac murmur may not be present. The chest pain that has been reported in patients with CAFs is due to a coronary steal phenomenon that results in a decrease of myocardial perfusion distal to the CAF [2]. Steal phenomenon is seen less often in CAFs that empty into the high pressure, low compliance LV as compared to fistulas that empty into the low pressure, high compliance right heart chambers. Further, blood flow through a CAF that empties into the right side of the heart is continuous during the cardiac cycle (systole-diastole), while blood flow in a CAF that empties into the LV occurs only during diastole; this difference is related to the different pressure and compliance between the LV and right ventricle.

For medical management of patients with multiple small CAFs, the factors determining myocardial oxygen supply and demand and the effects of pharmacologic agents on these factors should be considered. The major factors determining coronary blood flow and thus, myocardial oxygen supply are coronary artery resistance, coronary perfusion pressure, and diastolic time, while factors determining myocardial oxygen demand are heart rate, myocardial contractility, and left ventricular systolic wall tension that is determined by left ventricular pressure and volume (Laplace's law) [7]. Beta-blockers by decreasing heart rate and myocardial contractility decrease myocardial oxygen demand and thus, have been used in cases of CAFs that empty into the LV; however, beta-blockers also increase diastolic time (i.e., myocardial perfusion time), which in turn may increase the steal phenomenon (i.e., flow through the CAF during diastole) [2]. The net effect of such therapy would be the balance between the steal phenomenon and the decrease in myocardial oxygen demand. Therapy with calcium channel blockers or nitrates that may decrease myocardial oxygen demand by decreasing the peripheral vascular resistance also have been used in these patients. A decrease in peripheral vascular resistance, however, may result in a decrease in LV diastolic pressure; a decrease in LV diastolic pressure may increase the steal phenomenon (i.e., increase of flow through CAF during diastole) [7]. Thus, the balance between the major factors determining myocardial oxygen supply and demand should be considered when pharmacologic agents are used to treat patients with multiple small CAFs emptying into the LV.

The patient presented in this case was not treated because it was thought that he did not have myocardial ischemia and importantly because the benefit of current therapy in small CAFs that empty into the LV is questionable. Large CAFs that have increase flow and may produce high output heart failure and/or angina can be occluded by a transcatheter or surgical approach [2, 5]. Although surgical methods of closure are associated with low reported mortality and morbidity, transcatheter closure during cardiac catheterization has become the method of choice given its less invasive nature as compared to surgery. Many percutaneous catheter techniques have been implemented in practice like coils, detachable balloons, vascular plugs, polyvinyl alcohol foam, Amplatzer duct occluder, and others [8]. Effective and/or the best therapy for multiple small CAFs that empty into the LV remains to be defined. Establishing a registry of patients with CAFs that empty into the LV with long-term follow-up with no or on “empiric” therapy will assist in better defining the natural course of the disease and in establishing a potentially effective management.
